# Effect of baseline serum vitamin D level on symptom and medication scores of subcutaneous immunotherapy in children with mite allergy

**DOI:** 10.3389/fped.2022.1018549

**Published:** 2022-11-01

**Authors:** Xiaoxiao Jia, Hang Zheng, Xiumei Yan, Huan Dai, Qiangwei Xiang

**Affiliations:** Department of Pediatric Allergy and Immunology, The Second Affiliated Hospital and Yuying Children’s Hospital of Wenzhou Medical University, Wenzhou, China

**Keywords:** 1-25 (OH) D3, dermatophagoides pteronyssinus, dermatophagoides farina, allergy, subcutaneous immunotherapy

## Abstract

**Introduction:**

Allergen immunotherapy (AIT) is considered to be the only treatment that may change the natural process of allergic diseases. Subcutaneous immunotherapy (SCIT) is a type of allergen immunotherapy that is commonly used in clinical practice. However, SCIT has inconsistent effects on individuals, and it is yet unclear what factors affect therapeutic efficacy. In recent years, vitamin D levels have been speculated as a potential factor influencing SCIT efficacy.

**Objective:**

To investigate the effect of serum vitamin D level on the SCIT efficacy in children with allergic rhinitis and/or asthma caused by dust mite allergy.

**Methods:**

According to the panel consensus, children with asthma and/or allergic rhinitis who received SCIT were divided into the vitamin D deficiency group (<12 ng/ml), vitamin D insufficiency group (12–20 ng/ml), and vitamin D sufficiency group (>20 ng/ml). Serum 1–25(OH) D3, blood eosinophil, total IgE, dermatophagoides pteronyssinus (Dp), and dermatophagoides farina (Df) specific IgE (sIgE) were detected, and questionnaires of symptom and medication scores were collected before and after one year of treatment.

**Results:**

After one year of SCIT treatment, the symptom and medication score significantly decreased (*P *< 0.05), but there was no difference between the efficacy in different groups (*P *> 0.05). Our study found a statistical difference in Dp sIgE level between the vitamin D deficiency and the sufficiency groups (*P *= 0.024), and vitamin D levels become lower with children's growth (Y = −0.8981*X + 34.26, *P *= 0.0025).

**Conclusions:**

No difference was found between the efficacy of one-year SCIT and serum vitamin D levels based on symptom and medication scores. Nevertheless, higher vitamin D levels may be associated with a decreased indicator of Dp allergy.

## Introduction

In recent years, the prevalence of allergic rhinitis and asthma has remained high and caused a heavy social burden (1, 2). However, allergen immunotherapy (AIT) is considered the only disease-modifying treatment for type I allergy, including allergic rhinitis and asthma (3). Through high-dose allergen exposure, AIT induces dendritic cells to produce interleukin (IL)-10, IL-12, and IL-27, meanwhile stimulating regulatory T cells (Treg) and regulatory B (Breg) cells to produce immunoglobulin A (IgA), IgG and blocking IgG/IgG_4_ antibodies, thus to correct immune dysfunction caused by allergy, leading to the tolerance of allergens and improving clinical symptoms (4). Subcutaneous immunotherapy (SCIT) is a type of allergen immunotherapy that is commonly used in clinical practice. A large number of animal and clinical studies and meta-analyses have confirmed the efficacy and safety of SCIT (5–8). Nevertheless, there is a lack of ideal and effective immunological indicators for predicting the efficacy of subcutaneous immunotherapy.

Vitamin D is one of the immunomodulators. 1–25 (OH) D3 regulates immune function by inhibiting the differentiation and maturation of dendritic cells and enhances the frequency of two distinct populations of Treg cells: IL-10 secreting and Foxp3+ Treg cell (9, 10). Noticeably, dendritic cells, Treg cells, and IL-10 also play a significant role in the mechanism of allergen immunotherapy. Moreover, some studies have found that subcutaneous immunotherapy combined with vitamin D can enhance the therapeutic effect (11, 12). It has been reported that there is a significant correlation between vitamin D deficiency and the risk of asthma attack development in children (13). Accordingly, we further speculate if vitamin D levels in the body could affect the efficacy of immunotherapy. An epidemiological study showed that sufficient baseline serum vitamin D levels might enhance the efficacy of SCIT in adults with hay pollen allergic rhinitis (14). But for dust mite allergic children, the effect of vitamin D levels on SCIT has not been reported. Therefore, this study aimed to investigate the relationship between baseline serum vitamin D levels and the clinical efficacy of subcutaneous immunotherapy in children with dust mite allergic rhinitis and/or asthma.

## Materials and methods

### Study population

In the study, a total of 76 children with bronchial asthma and/or allergic rhinitis who received allergen specific immunotherapy were set as the observation group, and their serum vitamin D levels were detected. According to the global panel recommendations (15), the population was divided into three groups based on serum vitamin D levels: vitamin D deficiency group (<30 nmol/L, i.e., <12 ng/ml), vitamin D insufficiency group (30–50 nmol/L, i.e., 12–20 ng/ml) and vitamin D sufficiency group (>50 nmol/L, i.e., >20 ng/ml) (16). All the children were given the symptom scores and medication scores by filling in the questionnaire at two time points: before they were given specific immunotherapy and after one year of AIT treatment.

Inclusion criteria: (1) diagnosis of mild to moderate asthma and/or allergic rhinitis; (2) age 4–14 years old; (3) course of rhinitis or asthma >1 year; (4) determination of serum specific IgE antibody level >grade 3, dust mite was the main source of allergen; (5) treatment time >1 year, injection times >20 times.

Exclusion criteria: (1) severe asthma; (2) Use of beta-blockers; (3) cardiovascular dysfunction; (4) immune deficiency and/or chronic infectious diseases.

The study was approved by the medical ethical committee of The Second Affiliated Hospital and Yuying Children’s Hospital of Wenzhou Medical University, and written informed consent was obtained from the parent or legal guardian of the children.

### Subcutaneous immunotherapy administration regimens

According to different administration methods, allergen specific immunotherapy commonly used in clinical practice includes subcutaneous immunotherapy (SCIT) and sublingual immunotherapy. All participants included in this study received SCIT. House dust mite allergen preparation (Alutard sq Der p, ALK Abello company, Denmark) was used for subcutaneous injection. The course of injection was divided into initial treatment (dose increase) and maintenance treatment (dose maintenance). In the initial treatment stage, the allergen dose gradually increased from 100 SQ-U/ml to 100,000 SQ-U/ml. Children with bronchial asthma used conventional therapy. In the initial treatment stage, the injection is usually once a week, which takes 15 weeks. Cluster therapy (Rush immunotherapy, RIT) is used for children with simple allergic rhinitis, and the initial treatment stage is 7 weeks. After reaching the maintenance dose, the first injection was injected every 2 weeks, and the second injection was injected every 4 weeks. After that, according to the changes in the children’s condition, the interval of injection was 4–8 weeks. It is generally recommended that the children should be injected once every 6 weeks. The efficacy was evaluated after 1 year of treatment (after 20 injections).

### Asthma and allergic rhinitis definition

According to the Global Initiative for Asthma guideline, asthma was diagnosed by professional pediatric physicians based on the history of respiratory symptoms such as wheeze, cough, chest tightness, and shortness of breath that change over time and in intensity, as well as variable expiratory airflow limitation (17). Severe asthma was defined using the European Respiratory Society and the American Thoracic Society’s severe asthma definition (2014) (18).

Allergic rhinitis was defined as an inflammation of the nasal lining induced by inhaling something allergic, such as animal dander or pollen, with symptoms such as sneezing, nasal congestion, runny nose, post-nasal drip, and nasal itching (19).

### Allergenic index

Allergenic indexes were detected before entering the group.

(1) Peripheral blood eosinophil level: the count of eosinophils in peripheral blood was detected by an automatic hematology analyzer (produced by Shenzhen Mindray Pharmaceutical Co., Ltd.), and its value <0.45 * 10^9^/L was considered normal.
(2) Serum total IgE: peripheral venous blood was collected, and serum total IgE was determined by fluorescence enzyme-linked immunosorbent assay. The detection ranged from 0.00 IU/ml to 2,500 IU/ml.
(3) Serum specific IgE: serum sIgE was measured by fluoroimmunoassay technique (UniCAP, Pharmcia Diagnostics, Uppsala, Sweden). The detection range was 0.1–100 kul/L. The value was divided into 7 grades: <0.35 kua/L is grade 0, 0.35−<0.7 kua/L is grade 1, 0.7−<3.5 kua/L is grade 2, 3.5−<17.5 kua/L is grade 3, 17,5−<50 kua/L is grade 4, 50−<100 kua/L is grade 5, ≥100 is grade 6.

### Efficacy evaluation of subcutaneous immunotherapy

Before each allergen injection, patients were instructed to grade their symptoms retrospectively in the past week. Visual analog scales and Drug scores were evaluated for both rhinitis and asthma at the start of treatment and then every 2 or 3 months. SCIT is considered effective if the medication score of concomitant drugs decreases and the symptoms are well controlled after treatment (5, 6).

(1) Symptom scores assessment (20): the following symptoms were assessed and scored on a scale of 0 to 10: chest (cough, wheeze, breathlessness, chest tightness), nose (sneeze, blockage, and running), eye (itching, streaming, redness, and swelling), and mouth and throat (dryness and itching) symptoms.
(2) Medication scores assessment (21): it was totaled for the allowed rescue treatments: each oral antihistamines tablet (5 mg) was scored 1. Inhaled corticosteroids (200 µg budesonide or equivalent) were scored 1. Short-acting β-agonist (one application) was scored 1. Nasal corticosteroids (50 µg budesonide or equivalent) were scored 0.75. Nasal antihistamines (one application) were scored 0.25.

### Statistical analysis

Considering the response rate of allergic rhinitis to subcutaneous allergen immunotherapy (80%) using the following formula:

$$n=\frac{{\left(Z_{1-(\alpha /2)}\right)}^{2}\left\{P_{1}\left(1-P_{1}\right)\right\}}{d^{2}}$$

if *d* = 0.15p and *P* = 0.8, the minimum sample size to detect a response was estimated at 44 patients (14).

Statistical analysis was carried out by statistics software (SPSS 21). The data of normal distribution are described by arithmetic mean and 95% confidence interval. Nonnormal data were expressed as a 95% confidence interval or median and interquartile distance (IQR) after geometric mean and logarithmic transformation. Multiple comparisons of ANOVA were used for pairwise comparisons among the three groups. A covariance test was used to compare the symptom scores before and after treatment. Linear regression analysis was applied to analyze the association between vitamin D levels and age. Graphical representations were made by using the GraphPad Prism statistical package. For all tests, *P* < 0.05 was considered statistically significant.

## Results

### Characteristics of the study population

A total of 76 children participated in the study. And symptom and medication scores of 62 patients improved with an effective rate of 81.58% (62/76). Eventually, the 62 effective patients were selected for subsequent analysis. There were 6 participants in the vitamin D deficiency group, 36 participants in the vitamin D insufficiency group, and 20 participants in the vitamin D sufficiency group, with the effective rate of 75.00% (6/8), 80.00% (36/45), and 86.96% (20/23), respectively. The eosinophil count of the vitamin D deficiency group, vitamin D insufficiency group and vitamin D sufficiency group were 0.61*10^9^/L, 0.46*10^9^/L, and 0.46*10^9^/L, respectively (Table 1).

**Table 1:** Demographic data of the study population.

Variable	Mean ± SD/*n* (%)			
	Vit D deficiency group (<12 ng/ml)	Vit D insufficiency group (12–20 ng/ml)	Vit D sufficiency group (>20 ng/ml)	*P*-value
Vit D levels, ng/Ml	18.76 ± 0.80	24.98 ± 2.57	35.13 ± 3.63	
Ineffective, *n* = 14 (18.42)	2	9	3	
Effective, *n* = 62 (81.58)	6 (75.00)	36 (80.00)	20 (86.96)	
Male	2	22	16	
Female	4	14	4	
Age, years	8.67 ± 3.50	7.67 ± 2.52	5.85 ± 1.87	0.012
Serum total IgE, IU/ml	731.83 ± 532.08	811.83 ± 706.01	561.02 ± 535.15	0.380
Dp specific IgE, kua/L	98.57 ± 3.51	77.33 ± 29.48	66.55 ± 33.88	0.071
Df specific IgE, kua/L	82.95 ± 16.66	72.87 ± 32.52	63.36 ± 32.06	0.342
Blood eosinophil, 10^9^/L	0.61 ± 0.62	0.46 ± 0.29	0.46 ± 0.23	0.528

Vit D, vitamin D; Ig, immunoglobulin; Dp, Dermatophagoides pteronyssinus; Df, Dermatophagoides farina.

### Vitamin D levels and age

**Figure 1:**
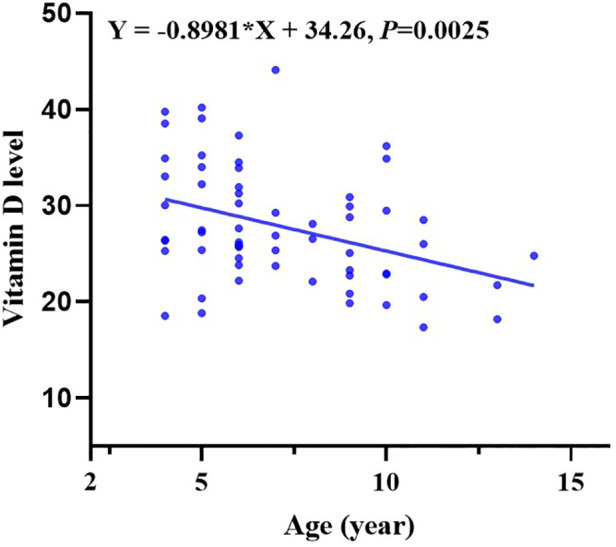
Vitamin D levels changed in different ages.

Moreover, multiple comparisons showed that the age in the vitamin D deficiency group, vitamin D insufficiency group, and vitamin D sufficiency group were statistically different with a *P* value <0.05 (Table 1). Vitamin D levels become lower with children’s growth. We further conducted a linear regression analysis and found a negative relationship between vitamin D levels and age with a regression coefficient (−0.8981) <0 and *P*-value <0.05 (Figure 1).

### Symptom score and medication score before and after AIT

**Figure 2:**
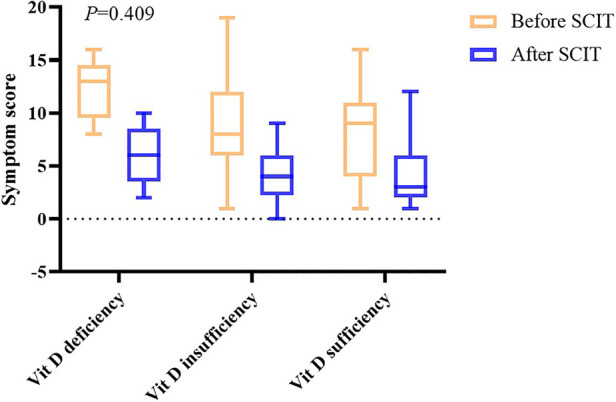
Symptom scores before and after AIT.

**Figure 3:**
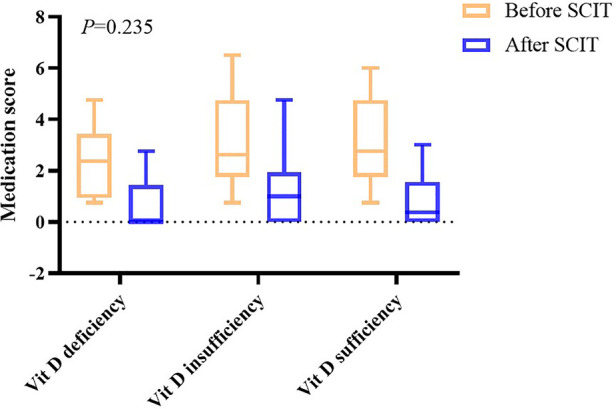
Medication scores before and after AIT.

After one year of AIT treatment, the symptom and medication scores statistically decreased (*P *< 0.05) (Table 2), but analysis of covariance showed there was no difference between the effectiveness in different groups (*P *= 0.409 in symptom scores and *P *= 0.235 in medication scores) (Figures 2, 3). Moreover, no difference was found between the symptom score of conventional therapy and cluster therapy (Supplementary Table S1).

**Table 2:** Symptom score and medication score during the immunotherapy.

Groups based on Vit D levels (ng/ml)	Before SCIT	After SCIT	*P*-value (efficacy)
Symptom score			
<12	12.33 ± 2.94	6.00 ± 2.83	0.0035*
12–20	8.58 ± 4.05	4.19 ± 2.34	<0.0001*
>20	8.37 ± 4.56	4.32 ± 3.20	0.0031*
Medication score			
<12	2.38 ± 1.47	0.63 ± 1.12	0.0427*
12–20	2.88 ± 1.78	1.22 ± 1.26	<0.0001*
>20	3.08 ± 1.73	0.76 ± 0.98	<0.0001*

Vit D, vitamin D.

The * in *P*-value (efficacy) indicates significant differences between the scores before and after SCIT (*P* < 0.05).

### Dp sIgE level and df sIgE level between groups

**Figure 4:**
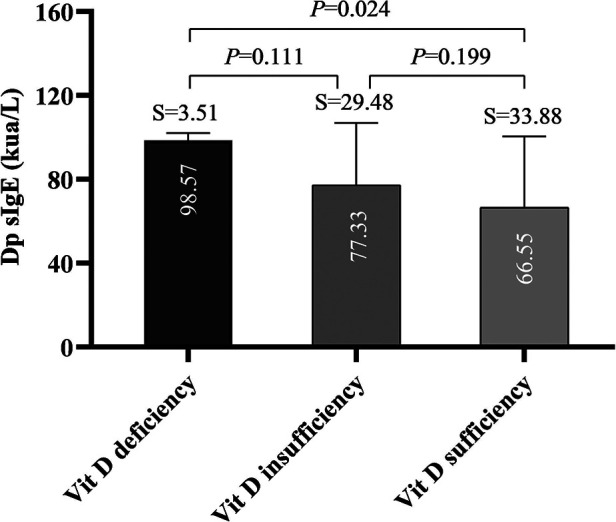
Dermatophagoides pteronyssinus (Dp) sIgE in different group.

Multiple comparisons among groups showed there was a significant difference in Dp SIgE level between the vitamin deficiency group and vitamin D sufficient group (*P *= 0.024), but no significant difference between the other two groups comparison (Figure 4). Nevertheless, we found no difference between Df sIgE levels in the vitamin D deficiency group, vitamin D insufficiency group, and vitamin D sufficiency group (*P* > 0.05) (Figure S1).

## Discussion

Our study suggested that subcutaneous immunotherapy could provide adequate relief of allergic rhinitis and asthma symptoms and improve medication scores. However, serum vitamin D levels were weakly associated with the efficacy of one-year subcutaneous immunotherapy. Still, vitamin D levels may be related to the decreased Dp sIgE levels in children receiving SCIT, which is one of the vital indicators for evaluating allergy.

The growing body of evidence indicated that vitamin D as an adjuvant combined with subcutaneous immunotherapy could enhance the efficacy of subcutaneous immunotherapy. Meanwhile, a recent epidemiological study suggested that baseline serum vitamin D levels were associated with subcutaneous immunotherapy in adults with hay pollen allergic rhinitis (14). Nevertheless, the effect of serum vitamin D levels on the subcutaneous immunotherapy of dust mite allergy in children has not been reported. According to the meta-analysis (at least 19 studies), lower vitamin D levels were correlated with a higher allergic rhinitis prevalence in children (22), and vitamin D affects not only the occurrence but also the course and treatment of asthma (23–25). Additionally, previous clinical studies found that in children with dust mite-induced asthma, vitamin D as an adjuvant participating in subcutaneous immunotherapy could achieve a better clinical effect compared to single subcutaneous immunotherapy (26). Experimental studies in mice have also demonstrated that adjuvant use of vitamin D may enhance the efficacy of subcutaneous immunotherapy in grass pollen-induced allergic asthma (11, 12). Vitamin D is an essential immune regulatory factor and may influence the development of asthma and allergy susceptibility through different mechanisms (27). Vitamin D affects innate immunity by inhibiting toll-like receptors and regulates acquired immunity by inhibiting T cell proliferation. Especially in the allergic state, vitamin D acts on the human immune system by inhibiting the growth cycle of human dendritic cells and regulating the function of T cells by increasing the secretion of IL-10 (27, 28). In addition, research has demonstrated that vitamin D is essential for maintaining the integrity of nasal mucosa epithelium, which makes it a potent defense against environmental allergens (27). From this, we further speculate that vitamin D levels in the body may affect the efficacy of immunotherapy through immune regulation.

Intriguingly, our study observed a substantial difference in the Dp SIgE level between the vitamin deficiency group and the vitamin D sufficiency group. Similarly, serum vitamin D levels are closely related to asthma and allergic diseases and were inversely correlated with serum total IgE levels (29). A prior study showed that long-term immune tolerance to allergens might be influenced by a decline in allergen-specific IgE concentrations (4). Therefore, this might mean that high vitamin D levels may not impair AIT’s effectiveness based on symptom and medication scores in our study, but they might make it easier for the body to develop long-term immunological tolerance.

In addition, this study also found that vitamin D deficiency becomes more severe with children’s growth, which is consistent with the previous research (30). Vitamin D levels are frequently influenced by diet and sun exposure. However, as information about diet and outdoor activities is lacking from our investigation, further epidemiological studies are needed to confirm the finding. Meanwhile, a meta-analysis reported that vitamin D levels had significant age-specific relations to the risk of aeroallergen sensitization and allergic rhinitis. Children with serum 25(OH)D ≥ 75 nmol/L were associated with decreased risk of aeroallergen sensitization, but vitamin D supplementation in infancy was not related to the risk of allergic rhinitis (31). Overall, our study suggests that although no correlation was found between serum vitamin D levels and the efficacy of specific immunotherapy, we should pay more attention to the vitamin D levels in school-age children and adolescents, especially in the allergy population.

Furthermore, this study has some limitations. The participants are all from Wenzhou, which does not accurately represent the country’s population. Further study is required to corroborate this finding because of the relatively small sample size and limited representation of participants.

## Conclusions

There were no differences between the efficacy of one-year allergen immunotherapy and serum vitamin D levels based on symptom scores and medication scores. We nevertheless discovered that lower vitamin D levels are linked to higher IgE levels of the dermatophagoides pteronyssinus allergy.

## Data Availability

The original contributions presented in the study are included in the article/**Supplementary Material**, further inquiries can be directed to the corresponding author/s.
