# Supplementary material for: Effect of baseline serum vitamin D level on symptom and medication scores of subcutaneous immunotherapy in children with mite allergy
Source: Front Pediatr. 2022 Nov 1;10:1018549. doi: 10.3389/fped.2022.1018549 (PMC9663479; doi:10.3389/fped.2022.1018549)
Supplement: Supplementary file 1 [file Datasheet1.docx]

Table S1. The comparisons between conventional therapy and cluster therapy

| Efficacy assessment | Conventional therapy | Cluster therapy | *P* value |
| --- | --- | --- | --- |
| Symptom score | 5.29±3.66 | 3.52±4.09 | 0.676 |

Figure S1. Dermatophagoides farina (Df) sIgE in different groups
